# Evaluating international collaboration on horizon scanning for pharmaceuticals: developing key performance indicators for the international horizon scanning initiative

**DOI:** 10.1017/S0266462325103358

**Published:** 2026-01-19

**Authors:** Brenda Leeneman, Hedwig M. Blommestein, Diana M.J. Delnoij, Maureen P.M.H. Rutten-van Mölken

**Affiliations:** 1 Erasmus School of Health Policy and Management, Erasmus University Rotterdam, the Netherlands; 2 Institute for Medical Technology Assessment, Erasmus University Rotterdam, the Netherlands; 3 National Health Care Institute, the Netherlands

**Keywords:** health policy, health technology assessment, horizon scanning, key performance indicator, pharmaceuticals

## Abstract

**Introduction:**

Many European countries independently conduct horizon scanning activities. However, the costs, time, and resources required can be prohibitive. To address this, the International Horizon Scanning Initiative (IHSI) was launched in 2019. IHSI aims to facilitate decision-makers and payers in negotiating fair prices and preparing for potentially disruptive pharmaceuticals. IHSI developed the Joint Horizon Scanning Database, a repository of pharmaceuticals expected to enter the European market, and initiated a series of High Impact Reports (HIRs) to highlight pharmaceuticals that have the potential to significantly impact healthcare systems. This paper outlines the development of key performance indicators (KPIs) that can be used to evaluate IHSI’s work.

**Results:**

In close collaboration with representatives from IHSI member countries and its Executive Committee, the following KPIs were developed: “Number of IHSI member countries”, “Embedding of IHSI in national health technology assessment procedures”, “Database coverage”, “Data completeness”, “Data timeliness”, “Accuracy of identifying disruptive pharmaceuticals”, “Accuracy of identifying non-disruptive pharmaceuticals”, “Use of HIRs in preparing for disruption to the healthcare system”, and “Use of HIRs in price negotiations and financial arrangements”. Among these, “Accuracy of identifying disruptive pharmaceuticals” was considered most important, followed by “Data timeliness” and “Data completeness”. Additionally, based on consultations with nonmember countries, strategies for improvement were identified should the KPIs reveal areas for improvement. These include involving patients in the selection of pharmaceuticals and conducting reputation surveys alongside measuring KPIs. While the KPIs and strategies for improvement are specific to IHSI, they can be tailored to support other (international) horizon scanning initiatives.

## Introduction

Technological and scientific advancements have substantially changed the pharmaceutical treatment landscape. While innovative therapies often improve health outcomes, their high costs impose considerable pressure on healthcare budgets. To ensure the efficient use of available resources, horizon scans of new pharmaceuticals have become increasingly important, as also acknowledged in the new Health Technology Assessment Regulation 2021/2282 (1;2). Horizon scanning enables healthcare decision-makers, payers, and providers to identify pharmaceuticals with a potentially significant impact on patients, public health, and healthcare systems at an early stage. This early identification is crucial for balancing timely access to innovative therapies with the financial sustainability of healthcare systems (1).

In Europe, many countries independently conduct horizon scanning activities. These activities allow countries to proactively anticipate potential applications for market access of these pharmaceuticals and to take early actions to mitigate their potential negative impact. However, the costs, time, and resources required for such activities can be prohibitive (1;3). In response to this, the International Horizon Scanning Initiative (IHSI) was launched in October 2019. Since then, nine European countries – Austria, Belgium, Denmark, Ireland, the Netherlands, Norway, Portugal, Sweden, and Switzerland – have become members of IHSI. By working together, IHSI aims to provide data that facilitate decision-makers and payers in negotiating fair prices and preparing for potentially disruptive pharmaceuticals (4). To this end, IHSI developed (together with its vendor ECRI) the Joint Horizon Scanning Database in 2022. This database serves as IHSI’s repository of new pharmaceuticals expected to enter the European market (5). Data on these pharmaceuticals are continuously collected through a combination of an automated process and human data curation. Additionally, IHSI initiated a series of High Impact Reports (HIRs) with the intention of highlighting pharmaceuticals that have the potential to significantly impact the healthcare systems of its member countries. The selection of pharmaceuticals included in these HIRs is based on input from five medical experts, each with at least 5 years of experience within their specialty.

All member countries collectively bear the costs of IHSI. Since these costs are financed with public resources, it is essential to demonstrate that the investments contribute to identifying new pharmaceuticals at an early stage, as well as providing relevant data to decision-makers and payers. Therefore, the aim of this study was to develop key performance indicators (KPIs) that can be used to evaluate IHSI’s work.

## Methods

The study was structured into four phases. First, a scoping review was conducted to obtain an overview of currently available KPIs related to horizon scanning for pharmaceuticals. Subsequently, using the information obtained from the review, interviews were performed with representatives from IHSI member countries to discuss the relevance and measurability of the identified KPIs. In addition, participants were asked to propose other potentially relevant KPIs that had not been identified through the scoping review. The KPIs that were considered relevant were then presented to the IHSI Executive Committee (EXCO), and participants were asked to rank them according to their perceived importance. Finally, to refine the KPIs, consultations were conducted with horizon scanning experts from nonmember countries.

### Scoping review

A Google search was performed (on July 10, 2023) using the following search strategy: “horizon scanning” AND “key performance indicators OR indicators” AND “pharmaceuticals OR medicines.” The search was limited to the first 50 hits. For each website returned by the search, multiple pages were reviewed to obtain information, specifically documents reporting KPIs related to horizon scanning for pharmaceuticals. In addition to the Google search, the Patients Waiting to Access Innovative Therapies (W.A.I.T.) Indicator (6) of the European Federation of Pharmaceutical Industries and Associations (EFPIA) was reviewed, which is a large study on the availability of pharmaceuticals in Europe.

### Interviews with member countries

Semi-structured interviews were conducted with representatives from seven member countries: Austria (N = 1), Belgium (N = 1), Ireland (N = 1), Norway (N = 2), Portugal (N = 1), Switzerland (N = 1), and the Netherlands (N = 3). The other two member countries, Denmark and Sweden, provided written input. All respondents were experienced and had senior level positions. Multiple topics were discussed during the interview, including the relevance of the KPIs identified through the scoping review, additional relevant KPIs, and the suitability of the Joint Horizon Scanning Database and the HIRs for measuring the KPIs. Prior to the interview, the participants received an overview of the questions. All responses were summarized, and the participants were asked to review and approve the summary of their responses.

### Priority setting

The results of the scoping review and interviews were presented at the EXCO meeting on November 22, 2023, including the KPIs considered relevant by the interviewees. During the presentation, the participants ranked the KPIs using an ordinal approach, indicating their perceived importance from highest to lowest.

### Consultations with nonmember countries

To gather additional insights, horizon scanning experts from nonmember countries were consulted. The following countries were selected: Australia, Canada, and the United Kingdom (UK), based on their experience with horizon scanning activities. The consultations focused on the use of KPIs in their respective countries to evaluate horizon scanning activities. All experts were asked to review and approve the summary of the insights gathered during the consultations.

#### Horizon scanning activities in Australia, Canada, and the UK

In Australia, there is currently no horizon scanning system at the national level. Instead, horizon scans are conducted independently by university-based HTA groups and professional societies (7). In Canada, horizon scans are performed by HTA agencies, such as Canada’s Drug Agency (CDA-AMC), primarily at the request of publicly funded pan-Canadian healthcare decision-makers and providers within the federal, provincial, and territorial jurisdictions. For each selected pharmaceutical, the CDA-AMC prepares a horizon scanning report, which contains a brief description of the pharmaceutical, trial information (including the clinical development phase), the target population, the setting in which the pharmaceutical is intended to be used, and the regulatory status in Canada (or elsewhere) (8). In the UK, a noncontractual agreement exists between the Department of Health and Social Care (representing the government), the National Health Service (NHS) England, and the Association of the British Pharmaceutical Industry (ABPI; representing the pharmaceutical industry), known as the Voluntary Scheme for Branded Medicines Pricing, Access, and Growth (VPAG). Under this agreement, the National Institute for Health and Care Excellence (NICE) is required to appraise all new pharmaceuticals entering the UK market and those with a significant new therapeutic indication. Horizon scanning is an essential step in the appraisals, as it provides NICE with clinical, financial, and service planning information about these pharmaceuticals (9). The UK’s national horizon scanning center, the National Institute for Health Research Innovation Observatory (NIHRIO), provides this information to NICE through technology briefings (10).

## Results

### Scoping review

#### Google search

The 50 websites returned by the Google search are listed in Supplementary Appendix 1. Only two websites referred to documents reporting relevant KPIs related to horizon scanning for pharmaceuticals: the website of the Italian Medicines Agency (AIFA) (11) and the website of Medicines Australia (12). The AIFA website made reference to a study by Marangi et al., (13) which described AIFA’s pharmaceutical horizon scanning system. In this study, the following KPIs were mentioned: time dedicated to activities, possible obstacles to the activities, and workloads. The website of Medicines Australia referred to a report by the European Network for Health Technology Assessment (14) in which recommendations were provided for horizon scanning. The following KPIs were mentioned in this report: timeliness, accuracy and completeness of data, public access to outputs, and usability of outputs for European cooperation on HTA. Both documents did not provide additional information on these KPIs. Therefore, the results from the scoping review were only used to support the development of a preliminary set of KPIs and were not used beyond this phase.

#### Patients W.A.I.T. Indicator

EFPIA’s Patients W.A.I.T. Indicator (6), introduced in 2004 and refined through evolving formats, evaluates the availability of innovative medicines and the time to patient access in European countries. Availability refers to whether a centrally approved medicine is included on a country’s reimbursement list. The current format consists of seven KPIs. Four of these KPIs are concerned with availability, measured by the number of medicines available to patients: total availability by approval year, rate of availability, rate of full availability, and breakdown of availability. The remaining three KPIs are related to the time to availability, measured by the time between the date of marketing authorization and the date of availability to patients: time from central approval (in the European Union) to availability, time to availability, and median time to availability (6).

### Interviews with member countries

The participants (n = 10) consisted of individuals with direct connections to IHSI, including founders and board members, as well as those with indirect affiliations. Half of the participants worked in health policy, primarily within ministries of health. The other half were involved in health technology assessment (HTA), focusing on the evaluation, pricing, and/or reimbursement of pharmaceuticals. All participants provided their consent for anonymously using their input and approved the summary of their responses. Given the importance of the Joint Horizon Scanning Database and the HIRs, the KPIs were categorized into general KPIs, database-related KPIs, and HIR-related KPIs. General KPIs include those without a direct connection to the database or HIRs.

#### General KPIs

Table 1 presents the set of general KPIs. One general, process-related KPI, defined as “Number of IHSI member countries”, was discussed with the participants. Although this KPI was considered to be informative, concerns were raised regarding its relevance as an indicator of impact. Becoming an IHSI member depends not only on IHSI’s expected impact but also on a country’s budget, as membership requires a minimum financial commitment. Furthermore, it depends whether a country has its own accurate horizon scanning system at the national level. Following suggestions from several participants, a second process-related KPI was included, which is defined as “Embedding of IHSI in national HTA procedures”. This indicator demonstrates the number of member countries that embedded IHSI as a formal step in their HTA procedure.

**Table 1.** Set of general KPIs

KPI: Number of IHSI member countries	
Explanation	This indicator demonstrates the number of IHSI member countries
Type of indicator	Process
Unit of measurement	Number

KPI: Embedding of IHSI in national HTA procedures	
Explanation	This indicator demonstrates the number of member countries that embedded IHSI as a formal step in their HTA procedure
Type of indicator	Process
Unit of measurement	Number

Abbreviations: HTA, health technology assessment; IHSI, International Horizon Scanning Initiative; KPI, key performance indicator.

#### Database-related KPIs

Prior to the interviews, two process-related KPIs with respect to the Joint Horizon Scanning Database were defined: “Data completeness” and “Data timeliness”. Both of these indicators were considered to be relevant. Based on input from the participants, we included a third process-related KPI, which is defined as “Database coverage.” The set of database-related KPIs is presented in Table 2.

**Table 2.** Set of database-related KPIs

KPI: Database coverage	
Explanation	This indicator assesses the overall completeness of the Joint Horizon Scanning Database
Numerator	The number of new pharmaceuticals and therapeutic indications approved by EMA that are included in the Joint Horizon Scanning Database
Denominator	The number of new pharmaceuticals and therapeutic indications approved by EMA
Type of indicator	Process
Unit of measurement	Proportion

KPI: Data completeness	
Explanation	This indicator assesses the completeness of data for each pharmaceutical in the Joint Horizon Scanning Database
Numerator	The number of pharmaceuticals for which complete data are available 1.5 years^a^ before the end of the pharmaceutical’s pivotal trial
Denominator	The number of pharmaceuticals in the Joint Horizon Scanning Database
Type of indicator	Process
Unit of measurement	Proportion

KPI: Data timeliness	
Explanation	This indicator demonstrates the time between completeness of data and the end of a pharmaceutical’s pivotal trial for each pharmaceutical in the Joint Horizon Scanning Database
Type of indicator	Process
Unit of measurement	Time in days, presented as the mean time across all pharmaceuticals

Abbreviations: EMA, European Medicines Agency; KPI, key performance indicator.

a This time period was selected by IHSI.

The KPI “Data completeness” assesses the completeness of data for each pharmaceutical within the database. More specifically, this indicator measures the proportion of pharmaceuticals for which complete data are available 1.5 years before the expected end of the pharmaceutical’s pivotal trial. This can be calculated by dividing the number of pharmaceuticals with complete data by the total number of pharmaceuticals in the database during a specific time period (e.g., annually). However, a clear definition of data completeness has not yet been established. We asked all participants to specify from the data dictionary which parameters should be available; two member countries (Austria and Denmark) provided input. Both countries indicated parameters such as a precise definition of the expected clinical indication, the prevalence, and the estimated total costs of the pharmaceutical. We also asked the participants of the EXCO meeting to share the five parameters that they consider most important. The same parameters – the indication, population size, and costs – were mentioned most frequently implying that these should be mandatory fields in the database. As the KPI “Data timeliness” demonstrates the time between achieving data completeness and the anticipated end of the pivotal trial, the definition of data completeness is also crucial for this indicator. For both KPIs, suggestions were made to differentiate between first-in-class and follow-on drugs, as well as between first and supplemental indications.

In addition to evaluating the completeness of data for individual pharmaceuticals, the KPI “Database coverage” assesses the overall completeness of the database. This can be quantified by dividing the number of new pharmaceuticals and therapeutic indications approved by the European Medicines Agency (EMA) that are included in the database by the total number of new pharmaceuticals and therapeutic indications approved by EMA during a specific time period (e.g., annually). Limiting to EMA approvals was considered appropriate, given the current member countries of IHSI.

#### HIR-related KPIs

In total, two process-related and five outcome-related KPIs with respect to the HIRs were discussed with the participants: “Accuracy of identifying disruptive pharmaceuticals” (process-related), “Accuracy of identifying non-disruptive pharmaceuticals” (process-related), “Use of HIRs in preparing for disruption to the healthcare system” (outcome-related), “Use of HIRs in price negotiations and financial arrangements” (outcome-related), “Time from central marketing authorization to availability” (outcome-related), “Restricted availability of pharmaceuticals” (outcome-related), and “Reduction in list prices of pharmaceuticals” (outcome-related). While both process-related KPIs were relevant and measurable, concerns were raised regarding the relevance and measurability of (some of) the outcome-related KPIs. For example, the time between central marketing authorization and availability to patients could depend on a pharmaceutical company’s launch strategy and/or country-specific regulatory processes, which cannot be influenced by IHSI. Additionally, since the majority of European countries use external reference pricing for setting the price of pharmaceuticals, IHSI will not affect prices of member countries that use nonmember countries as reference. Taking these concerns into account, we decided to continue only with the outcome-related KPIs that demonstrate the use of the HIRs. Table 3 presents the set of HIR-related KPIs.

**Table 3.** Set of HIR-related KPIs

KPI: Accuracy of identifying disruptive pharmaceuticals	
Explanation	This indicator evaluates IHSI’s ability to accurately identify pharmaceuticals with the potential to disrupt the key areas of healthcare, including patient outcomes, care delivery, healthcare infrastructure, patient access, and healthcare costs
Numerator	The number of pharmaceuticals that demonstrated to be disruptive after market entry^a^
Denominator	The number of identified disruptive pharmaceuticals
Type of indicator	Process
Unit of measurement	Proportion

KPI: Accuracy of identifying nondisruptive pharmaceuticals	
Explanation	This indicator evaluates IHSI’s ability to accurately identify pharmaceuticals that will not disrupt the key areas of healthcare, including patient outcomes, care delivery, healthcare infrastructure, patient access, and healthcare costs
Numerator	The number of pharmaceuticals that demonstrated not to be disruptive after market entry^a^
Denominator	The number of identified nondisruptive pharmaceuticals
Type of indicator	Process
Unit of measurement	Proportion

KPI: Use of HIRs in preparing for disruption to the healthcare system	
Explanation	This indicator demonstrates whether the HIRs are used to prepare for pharmaceuticals with the potential to disrupt the key areas of healthcare, including patient outcomes, care delivery, healthcare infrastructure, patient access, and healthcare costs
Type of indicator	Outcome
Unit of measurement	Descriptive

KPI: Use of HIRs in price negotiations and financial arrangements	
Explanation	This indicator demonstrates whether the HIRs are used in price negotiations and financial arrangements with pharmaceutical companies
Type of indicator	Outcome
Unit of measurement	Descriptive

Abbreviations: HIR, High Impact Report; IHSI, International Horizon Scanning Initiative; KPI, key performance indicator.

a The timing for conducting the reassessments is yet to be determined.

The process-related KPI “Accuracy of identifying disruptive pharmaceuticals” evaluates IHSI’s ability to accurately identify pharmaceuticals with the potential to disrupt the key areas of healthcare, including patient outcomes, care delivery, healthcare infrastructure, patient access, and healthcare costs. These pharmaceuticals are selected by medical experts, based on their potential impact score, and included in the HIRs. In general, pharmaceuticals are included if they receive a score of 2.2 or higher on a scale ranging from 0 (no impact) to 3 (high impact). By conducting (selective) reassessments, the proportion of accurately identified disruptive pharmaceuticals can be calculated by dividing the number of pharmaceuticals that demonstrated to be disruptive after market entry by the number of identified disruptive pharmaceuticals (i.e., all pharmaceuticals with an impact score of 2.2 or higher at the initial assessment). Ideally, disruptiveness after market entry should be determined using objective data, such as the number of patients who had access to the pharmaceutical, the number of patients treated with it, and its actual budget impact. When these data are not available, the reassessments could be based on subjective data, such as expert opinion. Note that the timing for conducting the reassessments is yet to be determined. The same approach can be applied to the process-related KPI “Accuracy of identifying non-disruptive pharmaceuticals”, but this time for pharmaceuticals with an impact score of less than 2.2.

The outcome-related KPI “Use of HIRs in preparing for disruption to the healthcare system” demonstrates whether the HIRs are used to prepare for (the market entry of) pharmaceuticals that might impact the aforementioned areas of healthcare. We explored the possibility to measure this KPI quantitatively, for example, by calculating the number of reports from agencies involved in preparing the healthcare system referencing the HIRs. However, due to the confidentiality of the HIRs and the complexity in determining the denominator, this approach was considered currently unfeasible. Some participants suggested alternative quantitative approaches, such as incorporating checkboxes into these reports or tracking the number of HIR downloads and views. Nevertheless, the majority of participants preferred a qualitative approach, such as interviews, for measuring this KPI. Stakeholders involved in preparing the healthcare system can be questioned by IHSI to obtain insight if, how, to what extent, and which aspects of the HIRs are used in the preparation. The same approach, that is, interviews with stakeholders involved in price negotiations and financial arrangements, is recommended for the outcome-related KPI “Use of HIRs in price negotiations and financial arrangements”, which demonstrates whether the HIRs are used in setting the price of pharmaceuticals.

### Priority setting

At the EXCO meeting, 18 participants ranked individually the nine KPIs described above. Ranks were converted into points, with the KPI ranked first receiving nine points and descending points assigned to KPIs ranked thereafter. KPIs that were not selected by a respondent received zero points. The points were summed to obtain a final ranking of the KPIs. The KPI “Accuracy of identifying disruptive pharmaceuticals” achieved the highest score (111 points), followed by the KPIs “Data timeliness” (88 points) and “Data completeness” (86 points). The lowest scores were observed for the KPIs “Accuracy of identifying non-disruptive pharmaceuticals” (36 points) and “Number of IHSI member countries” (37 points). The KPI “Data completeness” was most frequently ranked first, whereas the KPI “Accuracy of identifying disruptive pharmaceuticals” appeared most often in the ranking of the participants. The full ranking results and underlying responses are included in Supplementary Appendix 2.

### Consultations with nonmember countries

The consulted experts were employed at the University of Melbourne (Australia), the CDA-AMC, and NICE (the UK). All experts approved the summary outlining the insights gathered during the consultations.

Both the CDA-AMC and NICE use KPIs to evaluate their horizon scanning activities. However, these KPIs are confidential, and the results are generally not accessible to the public. Nevertheless, the consultations provided valuable insights into strategies that could be implemented to improve IHSI’s work, should the KPIs (we developed) reveal areas for improvement. The following strategies were identified: (i) assigning responsibility to pharmaceutical companies for entering and updating data in the Joint Horizon Scanning Database, (ii) involving patients in the selection of pharmaceuticals for inclusion in the HIRs, and (iii) conducting reputation surveys alongside measuring KPIs. These strategies were presented to IHSI’s Board of Directors for their feedback. The insights underlying the strategies, along with the feedback from IHSI’s Board of Directors, are elaborated upon below.

#### Assigning data responsibility to pharmaceutical companies

The strategy of assigning responsibility to pharmaceutical companies for entering and updating data in the Joint Horizon Scanning Database was based on insights gathered during the consultation with the expert from NICE. For NIHRIO, the primary source of information is the UK Pharmascan, which is a database containing information on new pharmaceuticals and indications in the pharmaceutical pipeline. Pharmaceutical companies enter data into this database from 3 years before UK availability or the start of phase 3 trials (whichever occurs first) up until availability in the UK. For each record (i.e., pharmaceutical or indication), the following information should be entered: data on the new pharmaceutical or indication, clinical trial data, regulatory data, and cost and budget impact data. Records are continuously updated as new information becomes available, and users, such as NIHRIO and NICE, are notified when updates occur (15). This strategy might alleviate IHSI’s responsibilities related to data collection and contribute to ensuring data completeness.

### Involving patients in the selection of pharmaceuticals

The strategy of involving patients in the selection of pharmaceuticals for inclusion in the HIRs was based on insights gathered during the consultation with the expert from the University of Melbourne. This expert developed a horizon scanning methodology that can be tailored to specific diseases and target populations. A key phase within this methodology is establishing consumer and clinician panels to define prioritization criteria for identifying pharmaceuticals that are most likely to impact the Australian healthcare system. In a case study, the methodology was applied to pharmaceuticals for colorectal cancer, melanoma, and non-small-cell lung cancer. The study showed differences in priorities between consumers and clinicians. Consumers prioritized toxicity and quality of life, with quality of life being the most important criterion in a curative setting and toxicity in a noncurative setting. In contrast, clinicians consistently prioritized survival, regardless of the setting (7). This strategy could improve IHSI’s ability to accurately identify potentially (non-)disruptive pharmaceuticals, as the study demonstrated that stakeholder perspectives (such as those of clinicians and consumers) affect perceptions of disruptiveness.

#### Conducting reputation surveys alongside measuring KPIs

The strategy of conducting reputation surveys alongside measuring KPIs was obtained from the CDA-AMC expert. In addition to evaluating horizon scanning activities through KPIs, the CDA-AMC administers annual reputation surveys to its stakeholders. These surveys were introduced to assess stakeholders’ perceptions of the CDA-AMC’s work and include questions regarding how often CDA-AMC’s services are used, the perceived usefulness of the services, trust in the organization’s work, the transparency of its procedures, areas for improvement, and the organization’s strengths and weaknesses.

#### Feedback from IHSI’s Board of Directors on the identified strategies

IHSI’s Board of Directors provided feedback on the identified strategies and expressed their interest in both involving patients and conducting reputation surveys. Involving patients or patient organizations had been discussed previously within IHSI but was not yet implemented due to various outstanding questions. For example, IHSI must first determine the appropriate approach for involving patients (either through national or cross-national patient organizations), decide on the optimal scope of their involvement, and consider whether and how to provide compensation. Regarding the reputation surveys, they suggest that these can be used to assess IHSI members’ perceptions of its activities. Insights gained from these surveys could complement those derived from the qualitative KPIs, which focus on the use of the HIRs by stakeholders involved in preparing the healthcare system, price negotiations, and financial arrangements. Together, these sources of information will provide a more comprehensive evaluation of how IHSI’s work is perceived. The strategy of assigning data responsibility to pharmaceutical companies is not considered viable, as IHSI has deliberately chosen to remain independent of company input and to rely on publicly available data in order to avoid confidentiality concerns.

## Discussion

This paper outlines the development of KPIs that can be used to evaluate IHSI’s work. In close collaboration with IHSI members, nine KPIs were developed: two general KPIs, three database-related KPIs, and four HIR-related KPIs. Additionally, three strategies for improvement were identified through consultations with nonmember countries, two of which could be implemented should the KPIs reveal areas for improvement. These strategies include involving patients in the selection of pharmaceuticals and conducting reputation surveys alongside measuring KPIs. The third strategy – assigning data responsibility to pharmaceutical companies – is not endorsed by IHSI, as maintaining independence and using only publicly available data is preferred. Although the developed KPIs and identified strategies for improvement are specific to IHSI, they can be tailored to support other (international) horizon scanning initiatives. Moreover, other publicly available sources on KPIs related to horizon scanning are currently scarce.

A key strength of this study is the comprehensive four-phase approach, including a scoping review, interviews with representatives from member countries, priority setting, and consultations with experts from nonmember countries, which enabled the development of relevant, measurable KPIs. However, even though scoping reviews are often conducted as a first step in a broader research process, several limitations of our review should be acknowledged. The scoping review covered scientific papers, grey literature, websites, and other sources, but the results depended on the selected search terms, and only the first 50 hits were assessed. Nevertheless, even a thorough review of the identified websites yielded only two relevant documents, suggesting that a broader search to include more hits or reviewing bibliographic databases would most likely not have led to additional results.

As previously noted, some KPIs require further refinement. This should be addressed before they can be used to evaluate IHSI’s work. For the database-related KPIs, the definition of data completeness is the most important aspect that needs refinement. Although we attempted to establish a clear definition by asking the participants of the interviews and EXCO meeting to specify the required parameters, additional input is necessary. The most frequently mentioned parameters – the indication, population size, and costs – still need further clarification. Therefore, we recommend identifying the stakeholders (or end-users) interested in the database and determining what information they request, when, and for what purpose. For example, for the parameter “indication”, potentially relevant subparameters are the target population, the stage of disease, the aim of the treatment (e.g., diagnostic, preventive, symptomatic, or curative), the place in therapy (e.g., first or second line), and whether the pharmaceutical will be used as monotherapy or in combination with another pharmaceutical (16). Two of the three most important KPIs (i.e., “Data completeness” and “Data timeliness”) both depend on a clear definition for data completeness. Therefore, establishing this definition, considering indication, population size, and costs is particularly relevant.

For the HIR-related KPIs, further refinement of the proposed reassessments is most important. These reassessments are part of the KPI “Accuracy of identifying disruptive pharmaceuticals”, which was ranked as most important by the IHSI EXCO. Timing of these reassessments should be determined and which experts to consult when objective data are not available. Ideally, these would be the same medical experts that conducted the initial assessment. When this is impracticable, the new panel of medical experts should align with the previous one, meaning that the panel should consist of five experts, each with at least 5 years of experience in the specific disease addressed by the pharmaceutical under review. Furthermore, it is important to understand that an impact score of less than 2.2 at the reassessment does not necessarily indicate inaccuracies in identifying disruptive pharmaceuticals. Information in the HIRs may have allowed countries to better prepare for the pharmaceuticals. Whether this is indeed the case can be discussed during the interviews which are recommended for measuring the KPI “Use of HIRs in preparing for disruption to the healthcare system”.

Furthermore, for the HIR-related KPIs with respect to the accuracy of identifying (non-)disruptive pharmaceuticals, we recommend involving patients. Patients can be involved in both the selection of pharmaceuticals for inclusion in the HIRs (i.e., the initial assessment) and in conducting the proposed reassessments (if objective data are not available) when improvements are required. As demonstrated by the study of the horizon scanning expert from Australia, including multi-stakeholder perspectives is likely to provide a more comprehensive understanding of the multifaceted impact of pharmaceuticals. Ultimately, this improves the preparation of healthcare systems for potentially disruptive pharmaceuticals.

Finally, in addition to evaluation IHSI’s work through KPIs, IHSI could consider introducing reputation surveys. As mentioned by the horizon scanning expert from Canada, the CDA-AMC uses these surveys to assess how stakeholders perceive its work. The introduction of similar surveys for IHSI would not only provide valuable insight into its stakeholders’ perceptions but also facilitate the identification of opportunities to further improve IHSI’s work.

## Conclusion

Developing KPIs to evaluate IHSI’s work is essential to demonstrate investments of member countries contribute to IHSI’s aim of providing data to facilitate decision-makers and payers in negotiating fair prices and preparing for disruptive pharmaceuticals. While the developed KPIs provide a solid foundation, further refinement is crucial for improving their measurability and providing an operational definition for data completeness should be a key priority.

## Supporting information

10.1017/S0266462325103358.sm001 Leeneman et al. supplementary material 1 Leeneman et al. supplementary material

10.1017/S0266462325103358.sm002 Leeneman et al. supplementary material 2 Leeneman et al. supplementary material

## Supplementary material

To view supplementary material for this article, please visit http://doi.org/10.1017/S0266462325103358.
