# Supplementary material for: Evaluating international collaboration on horizon scanning for pharmaceuticals: developing key performance indicators for the international horizon scanning initiative
Source: Int J Technol Assess Health Care. 2026 Jan 19;42(1):e10. doi: 10.1017/S0266462325103358 (PMC12902162; doi:10.1017/S0266462325103358)
Supplement: Leeneman et al. supplementary material 1 — Leeneman et al. supplementary material [file S0266462325103358sup001.docx]

# Appendix 1. Results of the Google search on KPIs related to horizon scanning for pharmaceuticals

| **Website** | **Relevant KPIs** |
| --- | --- |
| https://www.aifa.gov.it/en/horizon-scanning | - Time dedicated to activities - Possible obstacles to the activities - Workloads |
| https://www.ema.europa.eu/en/documents/report/emas-regulatory-science-strategy-2025-mid-point-achievements-end-2022_en.pdf | NA |
| https://www.gov.uk/government/publications/the-2019-voluntary-scheme-for-branded-medicines-pricing-and-access-operational-review-13-february-2023/13-february-2023-minutes | NA |
| https://www.linkedin.com/pulse/you-aware-innovative-medicines-fund-imf-offers-new-rare-richardson | NA |
| https://alexion.com/Documents/Alexion-IMF-infographic.pdf | NA |
| https://www.researchgate.net/figure/Different-levels-of-international-collaboration-in-the-procurement-of-medicines_fig2_320102519 | NA |
| https://www.england.nhs.uk/wp-content/uploads/2021/02/B0255-nhs-commercial-framework-for-new-medicines-response-to-engagement.pdf | NA |
| https://energy-shifts.eu/wp-content/uploads/2020/01/ApproachtoidentifyingfutureSSHenergyresearchpriorities.pdf | NA |
| https://www.rpharms.com/Portals/0/DRAFT%20Agenda%20Digital%20Pharmacy%20EAG%20Jan%202023_1.docx | NA |
| https://www.medicinesaustralia.com.au/publications/issues-briefs/national-medicines-policy/ | - Timeliness - Accuracy and completeness of data - Public access to outputs - Usability of outputs for European cooperation on HTA |
| https://www.health.nsw.gov.au/workforce/alliedhealth/Documents/pharmacy-horizons-scanning-report.pdf | NA |
| https://apps.who.int/gb/ebwha/pdf_files/WHA72/A72_17-en.pdf | NA |
| https://www.elft.nhs.uk/sites/default/files/2022-09/Appendix%203%20-%20JD%20Band%208b%20Lead%20Pharmacist%20BCHS.pdf | NA |
| https://www.health.gov.au/sites/default/files/documents/2022/04/review-of-the-quality-use-of-medicines-program-s-delivery-by-nps-medicinewise.pdf | NA |
| https://apply.jobs.scot.nhs.uk/Job/GetJobAdvertDocument?docid=394327&GUID=83033091-10b6-416d-9ad9-721c8631095d&jobid=91527 | NA |
| https://www.iqvia.com/-/media/iqvia/pdfs/library/white-papers/the-impact-of-biosimilar-competition-in-europe-2022.pdf | NA |
| https://www.nice.org.uk/Media/Default/accelerated-access-collaborative/AAC-Terms-of-Reference.pdf | NA |
| https://www.irishjobs.ie/job/senior-clinical-pharmacist/wallace-myers-international-job100578679 | NA |
| https://www.derbyshiremedicinesmanagement.nhs.uk/assets/japc/JAPC/Prescribing_spec_2023_2024.pdf | NA |
| https://www.edenscott.com/app/data/tmp/files/JD569%20-%20SMC%20Chief%20Pharmaceutical%20Adviser(1).pdf | NA |
| https://www.jobs.ie/ApplyForJob.aspx?Id=2147841 | NA |
| https://assets.publishing.service.gov.uk/government/uploads/system/uploads/attachment_data/file/447306/MHRA_Triennial_Review_Report.pdf | NA |
| https://www.blood.gov.au/pubs/0910report/chapter03/3.1.html | NA |
| https://potatoes.ahdb.org.uk/tags/blackheart | NA |
| https://beneluxa.org/sites/beneluxa.org/files/2017-07/BeneluxA_Terms_of_References_final_0.pdf | NA |
| https://vitaltransformation.com/page/3/ | NA |
| https://www.food.gov.uk/board-papers/fsa-science-update-2022 | NA |
| https://www.whatdotheyknow.com/request/463484/response/1109902/attach/html/4/Associate%20Director%20Medicines%20Optimisation%20JD.pdf.html | NA |
| https://pharmacoeconomics-congress.eu/doc/PHARMACA_Suppl_1_22FINALNAVERZIJA2.pdf | NA |
| https://medicinesauthority.gov.mt/file.aspx?f=5535 | NA |
| https://www.uhbristol.nhs.uk/media/3116116/17-515_attachment_combined_redacted.pdf | NA |
| https://www.stah.org/assets/Research-Centre/STAH_research_strategy_2018-2022.pdf | NA |
| https://www.mkuh.nhs.uk/document/response-4155-attachment-2 | NA |
| https://www.abpi.org.uk/media/cutbjd1e/homecare-mah-good-practice-guide-single-page-fiora-060123.pdf | NA |
| https://www.pbs.gov.au/general/medicines-industry-strategic-agreement-files/MA-Strategic-Agreement.DOCX | NA |
| https://www.lexisnexis.com/uk/lexispsl/financialservices/synopsis/135604:144726/Key-developments-and-horizon-scanning/Brexit?wa_origin=gnb | NA |
| https://publications.parliament.uk/pa/cm200607/cmhansrd/cm061214/text/61214w0028.htm | NA |
| https://www.enhertsccg.nhs.uk/sites/default/files/content_files/Gov_Body_July14_MIP/Item%2013.pdf | NA |
| https://www.cadth.ca/sites/default/files/corporate/planning_documents/2020-21-cadth-business-plan-final_update%20April%2016%202020.pdf | NA |
| https://www.apec.org/docs/default-source/groups/lsif/2019/final-report-of-the-2019-lsif-policy-dialogue-on-innovation-regulatory-systems-and-regulatory-conver.docx | NA |
| https://www.fmlm.ac.uk/aileen-parke | NA |
| https://hduhb.nhs.wales/about-us/governance-arrangements/board-committees/finance-committee/finance/finance-committee-meeting-13-march-2020/item-3-1-primary-care-prescribing | NA |
| https://www.elms-nfp.co.uk/website/X05413/files/v2%200%20-%20Prioritisation%20policy.docx | NA |
| https://www.gsk.com/media/9970/strategic-report-2022.pdf | NA |
| http://emig.org.uk/our-work/ | NA |
| https://www.ukri.org/publications/ukri-strategy-2022-to-2027/ukri-strategy-2022-to-2027/ | NA |
| https://www.tescoplc.com/media/759057/tesco-annual-report-2022.pdf | NA |
| https://www.cntw.nhs.uk/content/uploads/2017/02/Agenda-item-7-iii-Qtr-2-BAF-and-CRR-BOD-Sept-2016.pdf | NA |
| https://rxmagazine.org/a-strategy-for-delivering-efficiency-savings/ | NA |
| https://glostext.gloucestershire.gov.uk/documents/s23581/Performance%20report.pdf | NA |

Abbreviations: HTA, health technology assessment; KPI, key performance indicator; NA, not applicable.
