# Supplementary material for: Evaluating international collaboration on horizon scanning for pharmaceuticals: developing key performance indicators for the international horizon scanning initiative
Source: Int J Technol Assess Health Care. 2026 Jan 19;42(1):e10. doi: 10.1017/S0266462325103358 (PMC12902162; doi:10.1017/S0266462325103358)
Supplement: Leeneman et al. supplementary material 2 — Leeneman et al. supplementary material [file S0266462325103358sup002.docx]

# Appendix 2. Results of the KPI ranking by the Executive Committee

**Full ranking results**

| **Rank** | **KPI** |
| --- | --- |
| 1 | Accuracy of identifying disruptive pharmaceuticals |
| 2 | Data timeliness |
| 3 | Data completeness |
| 4 | Database coverage |
| 5 | Embedding of IHSI in national HTA procedures |
| 6 | Use of HIRs in price negotiations and financial arrangements |
| 7 | Use of HIRs in preparing for disruption to the healthcare system |
| 8 | Number of IHSI member countries |
| 9 | Accuracy of identifying non-disruptive pharmaceuticals |

Abbreviations: HIR, High Impact Report; HTA, health technology assessment; IHSI, International Horizon Scanning Initiative; KPI, key performance indicator.

**Underlying responses**

|  | **Rank** | | | | | | | | | **Score** |
| --- | --- | --- | --- | --- | --- | --- | --- | --- | --- | --- |
| **KPI** | **1st** | **2nd** | **3rd** | **4th** | **5th** | **6th** | **7th** | **8th** | **9th** |  |
| Number of IHSI member countries | 1 | 1 | 0 | 1 | 1 | 1 | 1 | 0 | 2 | 111 |
| Embedding of IHSI in national HTA procedures | 1 | 0 | 3 | 2 | 1 | 0 | 2 | 2 | 0 | 88 |
| Database coverage | 3 | 1 | 3 | 1 | 0 | 0 | 1 | 0 | 0 | 86 |
| Data completeness | 5 | 2 | 3 | 0 | 0 | 1 | 0 | 0 | 0 | 65 |
| Data timeliness | 3 | 2 | 0 | 6 | 1 | 1 | 0 | 0 | 0 | 57 |
| Accuracy of identifying disruptive pharmaceuticals | 2 | 8 | 3 | 0 | 0 | 2 | 0 | 0 | 0 | 56 |
| Accuracy of identifying non-disruptive pharmaceuticals | 0 | 1 | 0 | 1 | 3 | 0 | 1 | 1 | 2 | 44 |
| Use of HIRs in preparing for disruption to the healthcare system | 1 | 0 | 1 | 1 | 3 | 0 | 1 | 2 | 0 | 37 |
| Use of HIRs in price negotiations and financial arrangements | 2 | 0 | 2 | 1 | 2 | 1 | 0 | 1 | 2 | 36 |

Abbreviations: HIR, High Impact Report; HTA, health technology assessment; IHSI, International Horizon Scanning Initiative; KPI, key performance indicator.
